# Aspergillus Osteomyelitis Secondary to Chronic Necrotizing Pulmonary Aspergillosis in a Patient With Rheumatoid Arthritis

**DOI:** 10.7759/cureus.17774

**Published:** 2021-09-06

**Authors:** Eloy E Ordaya, James R Johnson, Dimitri M Drekonja, Gloria E Niehans, Anjum S Kaka

**Affiliations:** 1 Medicine, University of Minnesota, Minneapolis, USA; 2 Infectious Diseases, Minneapolis Veterans Affairs Health Care System, Minneapolis, USA; 3 Pathology and Laboratory Medicine Service, Minneapolis Veterans Affairs Health Care System, Minneapolis, USA

**Keywords:** aspergillus osteomyelitis, invasive fungal disease, immunocompromised hosts, rheumatoid arthritis, chronic necrotizing pulmonary aspergillosis, aspergillosis

## Abstract

*Aspergillus *spp. are ubiquitous molds that cause a wide range of clinical syndromes depending on the immune status of the host. Herein, we present a case of a patient with rheumatoid arthritis on long-term immunosuppressive medications, with a persistent dry cough and left-sided chest pain for over a year, who presented with acute sternal drainage. Computed tomography of the chest showed chronic pulmonary abnormalities, parasternal fluid, and bone destruction of the distal sternum and left sixth rib. The patient underwent debridement; sternal biopsy tissue showed septate hyphae with acute-angled branching, and *Aspergillus fumigatus* grew in culture. We suspected that the patient developed chronic necrotizing pulmonary aspergillosis (CNPA) that traversed tissue planes and caused chest wall osteomyelitis. The patient received voriconazole and surgical debridement, with clinical and radiological improvement. This case demonstrates the importance of considering CNPA as a diagnosis in patients with moderate degrees of immunosuppression and chronic respiratory symptoms, and *Aspergillus* spp. as an etiology of osteomyelitis in such patients.

## Introduction

*Aspergillus* spp. are ubiquitous filamentous fungi that exist in many environments, including air, soil, and decaying vegetation. Only a few of the approximately 200 recognized species of *Aspergillus* are human pathogens; of these, *A. fumigatus* is the most common [1]. *Aspergillus* spp. most often cause pulmonary disease, which presents as diverse syndromes that correlate with the immune status of the host [1]. By contrast, *Aspergillus* spp. uncommonly cause osteomyelitis and usually do so in immunocompromised patients. Bone involvement can be caused by direct trauma, prior surgery, hematogenous dissemination, or by direct invasion from the lung [2,3]. Here, we present the case of a patient with rheumatoid arthritis on long-term immunosuppression, with indolent respiratory symptoms, who subsequently developed sternal osteomyelitis due to *A. fumigatus*.

## Case presentation

A 77-year-old man with a history of long-standing rheumatoid arthritis with possible lung involvement, chronic obstructive pulmonary disease, asbestos-related pleuropulmonary disease, traumatic hydropneumothorax, controlled type-1 diabetes mellitus, and coronary artery bypass grafting (25 years ago), presented with two days of purulent drainage from his distal sternum. He reported having had a persistent dry cough and left-sided chest pain for over a year. Multiple computed tomograms (CTs) of the chest had shown stable left-sided pulmonary nodules with fibrotic changes and a post-traumatic hydropneumothorax. His pulmonary signs/symptoms and imaging findings had been attributed to rheumatoid lung disease and remote trauma. The patient had received rituximab for the past eight months but had previously also been treated with etanercept, methotrexate, leflunomide, and intermittent courses of corticosteroids. He denied fever, weight loss, or joint pain. The patient lived in Minnesota during the summer and Arizona during the winter. He denied any other travel, exposure to mold or dust, or pet ownership.

**Figure 1:**
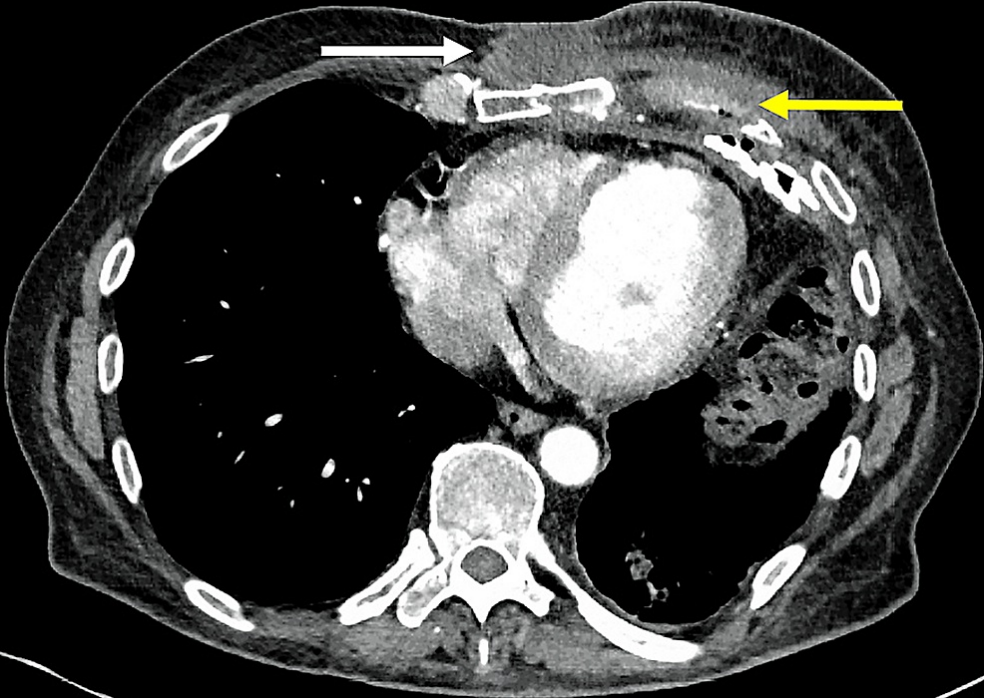
Computed tomography of the chest showing a parasternal fluid collection (white arrow) and osteolysis of the left sixth rib (yellow arrow).

On presentation, vital signs were normal. The only notable physical findings were a fluctuant swelling over the distal sternum, with a small sinus tract that drained copious amounts of cloudy brown fluid, and faint pre-sternal erythema. His white blood cell count was 7,300 cells/µL, alkaline phosphatase level 296 IU/L, C-reactive protein level 98 mg/L, and erythrocyte sedimentation rate >130 mm/h. A chest CT showed new bony destruction of the left sixth rib, with an adjacent parasternal fluid collection, but otherwise stable left-sided pleuropulmonary changes (Figure 1).

**Figure 2:**
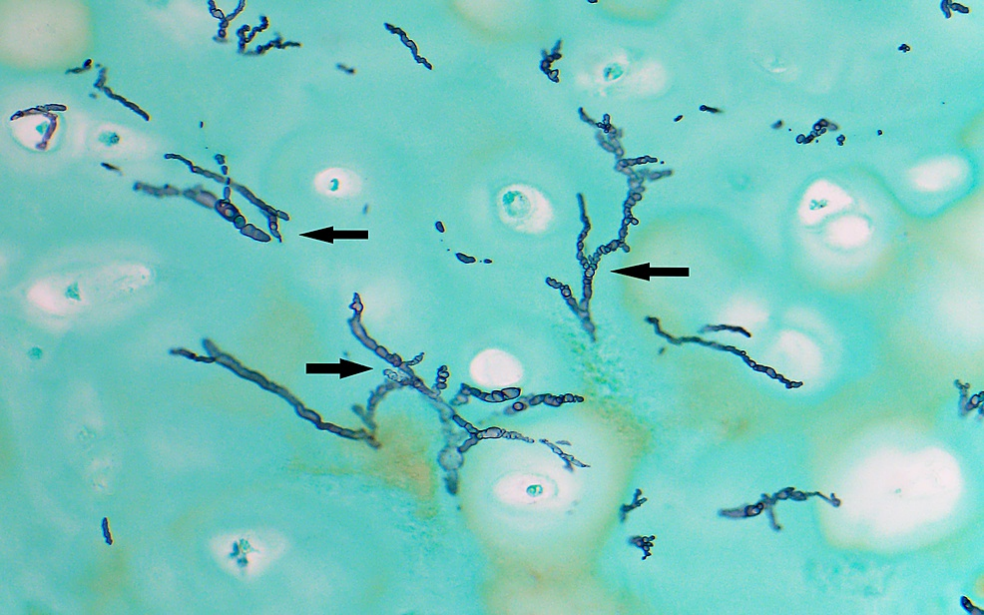
Histopathology of left sixth rib cartilage stained with Gomori methenamine silver (GMS) showing septate hyphae with acute-angle branching (arrows) (x50 magnification).

**Figure 3:**
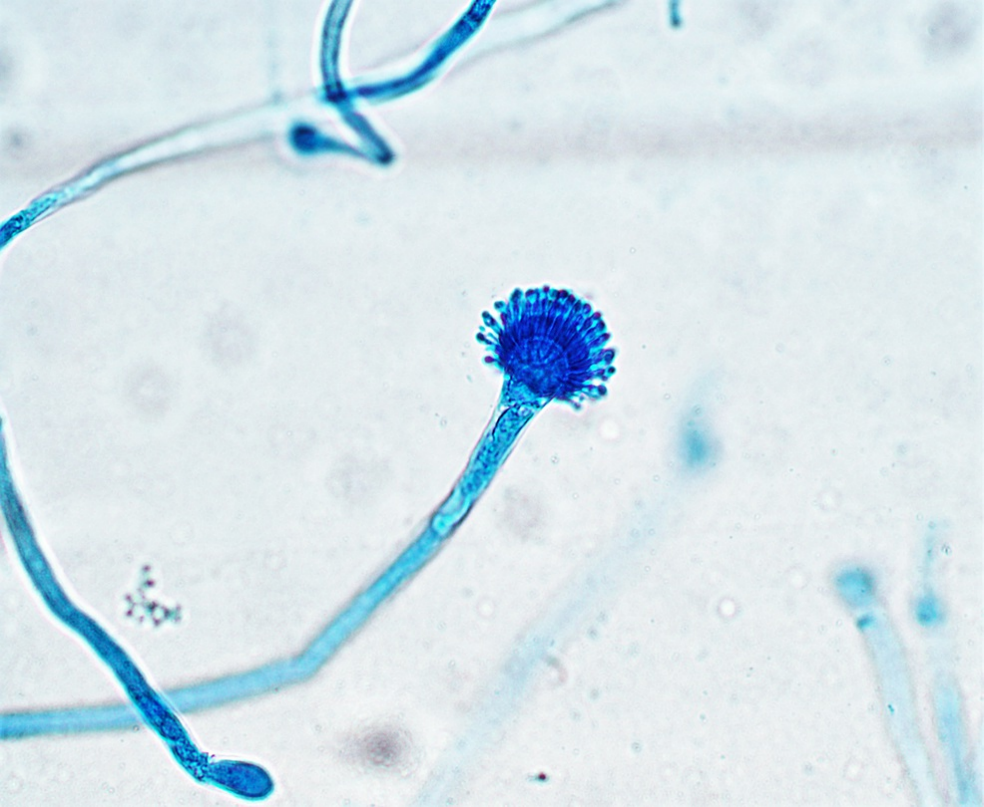
Fungal culture of left sixth rib cartilage showing Aspergillus fumigatus (x40 magnification).

The patient received vancomycin and piperacillin/tazobactam, and the following day underwent surgical debridement. Histopathological examination of the rib cartilage showed septate fungal hyphae with acute-angle branching consistent with *Aspergillus* spp. (Figure 2), and culture of the tissue yielded *A. fumigatus* (Figure 3).

The antibiotics and immunosuppressive therapy for rheumatoid arthritis were stopped, and the patient received oral voriconazole and underwent aggressive debridement of bone and soft tissues. After four months of voriconazole therapy, the sternal wound was healing, and repeat chest CT showed stable pulmonary and bone changes. Unfortunately, shortly thereafter, the patient died of unknown causes.

## Discussion

Pulmonary infection by *Aspergillus* spp. is acquired by inhalation of airborne spores and its clinical presentation is determined by the host immune response [4]. It ranges in severity from aspergilloma (a fungus ball that develops in a pre-existing pulmonary cavity, with little to no tissue invasion; hosts lack significant immune deficits) to invasive pulmonary aspergillosis (rapidly destructive pulmonary disease, sometimes with dissemination; hosts have severe immune deficits) [4]. Chronic necrotizing pulmonary aspergillosis (CNPA) falls between these extremes, affecting patients with mild-to-moderate immune deficits and pre-existing pulmonary disorders [1,4]. As in the present case, CNPA manifests as a locally invasive disease without dissemination that progresses over months to years [1,5].

Our patient first developed respiratory symptoms while receiving etanercept and intermittent corticosteroids courses, and experienced disease progression with rituximab therapy. Etanercept is a TNF-α inhibitor used for treating rheumatoid arthritis that increases the risk for mycobacterial and fungal infections, including aspergillosis, by impairing granuloma formation and neutrophil function [6-9]. Rituximab, an anti-CD20 monoclonal antibody, leads to B-cell depletion but has not been associated with an increased risk of aspergillosis compared to other biologic agents [10-12]. Corticosteroids affect virtually all aspects of the humoral and cellular immune response and predispose to different forms of aspergillosis [13,14]; low-dose corticosteroids use is a risk factor for CNPA [1].

We hypothesize that our patient initially developed CNPA of the left lung that insidiously (over > 6 months) traversed tissue planes to cause sternal and costal osteomyelitis. His multiple comorbidities, both pulmonary (chronic obstructive pulmonary disease, asbestosis, and possible rheumatoid lung disease) and systemic (rheumatoid arthritis and diabetes mellitus), combined with his multiple immunosuppressive medications (etanercept and corticosteroids), increased his risk for this invasive form of chronic aspergillosis.

Although osteomyelitis due to *Aspergillus* spp. is uncommon, contiguous spread to the bone from a pulmonary focus has been described, mainly in patients with diabetes, chronic granulomatous disease, malignancies, or corticosteroids therapy [2,3,15]. *Aspergillus* osteomyelitis has an indolent presentation. Pain and tenderness are the predominant symptoms (80%) [3], whereas sinus tracts with purulent drainage (27%) and fever (≤20%) are comparatively uncommon [16]. Inflammatory markers are frequently elevated. Diagnosis is established by biopsy and/or culture [3]. Treatment consists of voriconazole for a minimum of 8 weeks (often longer) along with surgical debridement. As in other types of aspergillosis, the reversal or reduction of immunosuppression should be attempted [17].

## Conclusions

CNPA affects patients with mild to moderate immune defects. Clinically, it is characterized by an insidious progression of pulmonary destruction that may involve contiguous structures. The typical paucity of systemic signs and symptoms may delay diagnosis. *Aspergillus* spp. are uncommon causes of osteomyelitis, and their treatment consists of surgical resection, antifungals, and reversal of immune suppression (to the extent possible).
